# Supplementary material for: Establishing the content of gender stereotypes across development
Source: PLoS One. 2022 Jul 12;17(7):e0263217. doi: 10.1371/journal.pone.0263217 (PMC9275684; doi:10.1371/journal.pone.0263217)
Supplement: Attachment — Submitted filename: Response to Reviewers.docx [file pone.0263217.s001.docx]

Dear Dr. Sokolov,

Thank you for the opportunity to revise and resubmit our manuscript, “Establishing the Content of Gender Stereotypes Across Development” (Manuscript #PONE-D-20-25142). My co-authors and I are delighted that the paper will be further considered for publication in *PLOS One.*

We have substantially revised the manuscript to address the excellent comments offered by you and the two anonymous reviewers. We believe that your collective feedback allowed us to significantly improve the paper, and we are grateful for your insights. A detailed list of our responses to your comments follows below the signature in this letter. Among many other improvements, we emphasize the following three major areas of revision.:

1.	First, the reviewers wanted additional clarity about the distinction between our study and previous work that has aimed to characterize the developmental trajectory for gender stereotypes (e.g., Koenig, 2018). We have highlighted some of the important methodological differences between these two studies. Most critically, our study elicited ratings for targets of particular ages (e.g., “seven-year-old boy”), instead of large ranges (e.g., “elementary school boys”; Koenig et al., 2018), allowing us to precisely graph and model developmental trajectories for particular stereotypes. This is important because a key question in developmental psychology is whether changes are continuous or discontinuous, and when they emerge. In order to address these compelling issues, a more fine-grained manipulation of target ages was necessary. In addition, we elicited ratings for individual traits (e.g., “is a leader”) instead of clusters of traits (e.g., “Agentic category: assertive, competitive, achievement-oriented, leadership ability”; Koenig, 2018). Our theories of the development of gender stereotyping will be different depending on the developmental trajectories of each separate stereotype. For example, we asked for ratings of 10-year-olds, 13-year-olds, and 16-year-olds, and one of our main findings is that there are large and potentially puberty-related changes in some (but not all) gender stereotypes that emerge and are strengthened between these years; this type of trajectory information is missing from previous work (e.g., Koenig, 2018), which asked for ratings of “elementary-aged children (5-12 years)” and “adolescents (12-18 years)”.

2.	Second, reviewers requested additional clarification on a host of minor methodological and terminological choices that were easily addressed by more clearly connecting our work to previous literature. We have made a number of minor changes throughout the manuscript, all of which are addressed in the response letter below.

3.	Finally, reviewers requested clarification about which elements of the analyses were preregistered and which were exploratory, and clarification about some of the analytic approaches we made. We have reorganized elements of our Results section to make our approach more clear (e.g., pg. 10), and have added clarification throughout. To be clear, with the exception of the clustering of our visualizations (which were qualitative and exploratory), all elements of our analyses were preregistered.

Thank you again for your consideration of this manuscript. We are grateful for the enthusiasm that you and the reviewers expressed for the current research (in addition to the valuable constructive feedback), as we believe that our paper examines an important yet under-explored topic. Please do not hesitate to contact me if there is anything that I can clarify, or if you feel that there are additional points that should be addressed. We sincerely hope you will feel that this significantly revised version of the manuscript is worthy of publication in *PLOS One* and look forward to receiving your feedback.

Sincerely,

The authors (blinded for review)

**Editorial Comments**

1. In your revision, please pay particular attention to the technical and statistical aspects of your work as mentioned in the Reviewers' comments.

**We have carefully attended to the reviewer’s technical and statistical comments, as detailed in our responses below.**

2. Also, statements of study primacy (such as "this is the first study..") have to be well-grounded. So, please make sure to justify the original rationale of your work and discuss your work and findings in the context of existing literature such as Koenig (2018), making clear the novelty of your research.

**We have carefully revised the entirety of the manuscript to ensure that claims of primacy are robustly grounded, given the state of the existing literature. Please also see response to comment #5 below.**

3. Please ensure that your manuscript meets PLOS ONE's style requirements, including those for file naming.

**We have ensured that our manuscript meets the journal’s style requirements, including those pertaining to file naming.**

**Reviewer #1**

4. I found this paper, which described gender stereotypes across childhood and adolescence, interesting and well-written.

**We thank the reviewer for their time, and for their kind words.**

5. I would like to see the method and results of this work more directly compared to those of past studies that cataloged prescriptive and descriptive stereotypes – including Koenig (2018, who also assessed prescriptive and descriptive stereotypes across age categories, very similar to the current study) as well as Prentice (in terms of the results for adults). I believe this study adds information above these past studies, but it would be helpful if the method and results were compared to highlight how this study extends and builds on this past research.

**We thank the reviewer for highlighting the usefulness of more directly comparing and contrasting our work to prior work published by Koenig (2018) and Prentice & Carranza (2002). We have highlighted the methodological differences between our study and previous work more clearly in our Method section, and, when possible, in the Results section.**

6. For example, I found it odd that the focus in the intro is on the stereotype content model for adults (pg. 11), which is about descriptive stereotypes, rather than this past work on prescriptive and descriptive stereotypes.

**We agree -- we have reduced our focus on the Stereotype Content Model in order to ensure that our literature review does not send readers down the wrong path. However, we do retain a brief reference to this model as it reflects one of the primary existing bodies of work on the nature of stereotypes about adults.**

7. I applaud the researchers for preregistering the study, but I was unclear what exactly was preregistered. The first time preregistration is mentioned on page 13 says “preregistered yet exploratory findings” but it is unclear at this point in the paper what these exploratory analyses would be. It would be useful here to outline the three specific goals currently listed later on page 16, which would help guide the reader and understand what was preregistered.

**We have now moved the bullet points as requested and have clarified the contents of our pre-registration. Further, we wish to clarify that in our labs we pre-register all studies as a means of adhering to best practices for promoting open sciences and reproducibility. We nevertheless consider our pre-registered analyses for this particular paper to be exploratory in the sense that we had no particular theoretical predictions with respect to possible outcomes. Because we fear that the term “exploratory” may be misunderstood to mean “unplanned” in this context, we have removed it and have clarified that all analyses were conducted as pre-registered.**

8. How was the list of traits created? Some of the traits seems to apply better to children or to adults. What were participants to do when rating whether adults wear tutus (unlikely) or “gets pushed around by other kids” (did this say “other adults” when rating adults?), for example?

**We have now clarified how we created the list of traits. We selected traits from Sullivan et al., 2018 Prentice & Carrana, 2002; and Rudman et al., 2012.**

9. It might not be possible for space reasons, but it would be quite helpful if the graphs of the different “visualization pattern” of stereotypes over time were incorporated into the table listing the stereotypes with different patterns, as it is difficult to remember the various patterns. Perhaps one prototypical stereotype could be directly graphed as an example, rather than (what I assume are currently) hypothetical patterns.

**We thank the reviewer for this thoughtful suggestion. We tried (several times, in fact). It simply won’t fit although we agree that it would be preferable to combine the table and figures. Given the online format of the journal, it seems possible that a hybrid table/figure could be possible in a web-format, even if it is not possible using Word Processing tools. if this paper is accepted and the editorial office wishes, we would be happy to work with the editorial office to explore the possibility of combining the figure and table (or interlinking them using hyperlinks) to allow for easier cross-referencing.**

10. It was unclear how it was decided which characteristics fit each pattern – was this a visual inspection by the researchers (and if so, was this done individually and then the labels compared, as is done for qualitative codings?) or was there a quantitative analysis of similarity (or whether the effect was linear or quadratic, etc.)?

**The creation of clusters, as noted in the manuscript, were planned, yet exploratory. We developed our coding scheme iteratively with one author (JS) clustering the patterns and the other two (AC and CMR) checking and commenting on the clusters. We do not wish to argue that these patterns are definitive but rather sought to find a digestible way to communicate the gender by age interactions that emerged in our dataset. We have clarified the process for generating these clusters (p. 18) and that the resulting clusters should be viewed as preliminary, stating “Due to the subjective and qualitative nature of this classification process, the resulting clusters should be interpreted as useful ways of digesting our otherwise exceptionally dense dataset, and as helpful jumping-off points for future research.”.**

11. Also, the characteristic “childlike” is listed twice in Table 6 under two different patterns.

**We have fixed this error -- “childlike” should only have been listed under “differences emerge late”.**

12. I would also have liked more discussion of the findings in terms of, for example, the types/groups of traits that showed different patterns or results. Are there common themes (e.g., communion, agency, negative communion, negative agency, competence, physical characteristics, personality traits vs. behaviors) in the traits that show different results? This would make it a bit easier to digest the findings, as well as having the individual traits listed out for the specifics.

**We thank the reviewer for this suggestion. In the Discussion section, we review some initial patterns that emerged in our reading of the current results (pp. 31-32). However, we wish to emphasize that, as noted clearly in our pre-registration and in the manuscript, these classifications are largely exploratory, and our dataset as a whole was intended to generate a large novel database that could be used to spark additional theory-building work. As such, we view any attempts to draw overarching conclusions regarding the patterns embedded in these results as currently premature. We hope that other researchers will find our data to be generative and will conduct additional confirmatory work (with an eye towards proposing eventual models of gender stereotyping across the lifespan).**

**Reviewer #2**

13. This manuscript reports a study of gender stereotypes across a variety of ages. This study appears to have been well-conducted overall. I have a few comments for the authors to consider as they move forward.

**We thank the reviewer for their thoughtful and supportive comments.**

14. In several places, the manuscript refers to effects over "the lifespan" or "the developmental timespan," but 6 of the seven ages are children/adolescents, and the other one, 35, is used as a stand in for all adults. Truly examining this over the lifespan would sample more widely across adulthood and include older adult ages (e.g., 65, 85). Please reframe the description of these effects to more accurately reflect the represented ages.

**Thank you for this comment -- we have been more careful to highlight what we mean by “the lifespan” and in many cases have changed this phrase to “across development”, including in the title of the manuscript.**

15. Relatedly, for Figure 2, the caption says that the data suggest that children may be subjected to the most gender stereotypes, but again you have six ages for children and only one for all of adulthood! I think this is quite a stretch to say given the data.

**We have clarified the justification for these claims. First, it is important to note that we did not collapse our data across ages, and therefore the fact that we sampled more child-targets than adult-targets does not impact this conclusion: for female targets, there were more stereotypes at age 7 than in adulthood; there were more stereotypes at age 10 than in adulthood; there were more stereotypes at age 13 than in adulthood; and there were more stereotypes at age 16 than in adulthood. As we now clarify this fact in the manuscript (pg. 25), this is particularly striking because much of the research to date has focused solely on adults, even though these data suggest that teenagers and adolescents may be subject to the largest number of stereotypes.**

16. It's unclear to me what "desirable" attributes are, as participants respond to these items. Are they saying they personally feel these attributes are desirable, or that they are seen in general as desirable? Do the authors have any validity evidence from this or other work using this wording that can tell us how best to understand how participants likely interpret "desirable" in this context? Literature on cultural vs. personal stereotypes would also be relevant to reference here.

**There are a couple of ways to address this, all of which we have adopted. The first is to be much more clear about the wording of the question that participants were asked. Specifically, they were asked “Indicate how desirable it is in American society for [age,gender] to possess each of the following characteristics. [Scale = 1-9; 1 = Not at all Desirable; 9 = Very Desirable]”.**

**Second, we have added the following footnote, which we hope addresses the reviewer’s concerns: “**We wish to note, of course, that “desirability” is a complex construct, and certainly not a construct that is likely to be fully addressed via a single likert-scale question. We use this terminology in order to most closely approach the ways in which previous work (e.g., Prentice & Carranza, 2002) has discussed prescriptive and proscriptive gender stereotypes.”

**Finally, while we have not included this in the main manuscript, we suspect that the reviewer may be concerned that the construct of “desirability” as, complex, context-dependent, and overly vague. In general, we agree. The main purpose of this paper was to adapt questions and methods that are typically used to ask about gender stereotypes about adults in order to ask these same questions about children. To that end, we use the methods, wording, and frameworks that have been relied on in previous work. Of course, desirability neither exists in a vacuum nor represents a unitary construct. In this paper, therefore, we use it merely to refer to the wording that participants responded to.**

17. The manuscript referred to preregistrations, which is great, but when I looked on OSF to see them I couldn't find them. This may be due to OSF's admittedly clunky navigation system (or my clunky inability to navigate it) but please do ensure that the preregistrations are made available.

**Thank you. The preregistration is available on OSF by clicking on “Registrations” on the top menu of our OSF page (https://osf.io/6r4ce/?view_only=5864111697d5441596520798ab675521). We have submitted a blinded link (which is a bit harder to navigate) for review. Should the manuscript be accepted, we will add an unblinded link (which is easier to navigate).**

18. The description of the analytical approach comes a little late - in the "Ratings of Typicality" section, but it looks like it was already employed to evaluate the "items that were not stereotyped" section. Please introduce the approach earlier to give context for how to interpret all reported results.

**Thank you -- we have reordered the results section in order to make this more clear.**

19. The Mturk sample is very large, which is great, but Mturk skews very White and likely other ways as well (e.g., politically liberal). Given that the stated aim is in part to create normed ratings of stereotypes, this should be explicitly acknowledged and discussed in the Discussion section.

**We have now added a clear statement indicating that we did not collect demographic data and therefore cannot assess the extent to which our sample represents the broader U.S. population.**

20. There is some ambiguity about what analysis was performed, exactly (e.g., for "stereotypes that change over the developmental timespan," did the authors use regression to identify interactions, or only the descriptive method described in this paragraph?). Explicitly and clearly describing all analyses would be helpful. For this reason I chose "I don't know" about whether the analyses have been performed appropriately and rigorously, but with just a little more explanation, I would answer "Yes."

**We did use regression techniques to identify significant interactions; we have reordered the content to make the structure of our analyses obvious. We have also added further clarification of our analytic technique (see p. 10).**

21. I particularly appreciate the focus on effect sizes. Could the authors explain their thinking behind choosing these particular cutoffs (e.g., why does a rating of 6 or higher count).

**We now clarify that we pre-registered these criteria in keeping with previous research (Prentice & Carranza, 2002). Further, we selected these specific cutoffs to be consistent with prior work that has explored stereotype content for adult targets (e.g., Rudman et al., 2012).**

22. Social roles theory of gender stereotypes seems relevant here, especially with respect to its focus on agency and communion, which are close to warmth and competence. I would encourage the authors to reference this work if/as relevant.

**As described in our response to comment #7, reviewer #1’s comment prompted us to reconsider whether our reliance on the stereotype content model was necessary or relevant for the purposes of the current work. As detailed above, we have limited this discussion, and now focus directly on distinguishing between descriptive, prescriptive, and proscriptive stereotypes across the lifespan (in keeping with prior work on backlash theory; e.g., Rudman et al., 2012a). Further, we now add a reference to social role theory (pp. 4-5, 6). However, we wish to reiterate that we view the primary goal of the current work as providing a fine-grained analysis of the content of descriptive, prescriptive, and proscriptive stereotypes across development. As such, we view it as premature to attempt to modify an existing theory of adult stereotyping for use with children and/or develop a new model of stereotyping of children -- although we agree that these are important long-term goals, and as such are issues we hope to address in the coming years. In sum, while we do briefly ground our discussion of the current work in existing theoretical perspectives, we hope that our data provide empirical fodder for the development of gender stereotyping models that apply across the developmental timespan.**

23. The approach taken here (using established traits from literature on gender stereotypes) makes sense. For their future work, I would encourage the authors to consider whether a bottom-up approach could complement this work, as that could allow them to see if there are unique gender stereotypes that arise at different ages that are not well-captured by existing gender stereotypes (which could reflect only certain ages).

**Absolutely! We love this idea!**
