# Supplementary material for: Establishing the content of gender stereotypes across development
Source: PLoS One. 2022 Jul 12;17(7):e0263217. doi: 10.1371/journal.pone.0263217 (PMC9275684; doi:10.1371/journal.pone.0263217)
Supplement: Attachment — Submitted filename: DevTraj_Reponse_Sept.docx [file pone.0263217.s002.docx]

**Editorial Comments**

**1) As you will see from the comments, both Reviewers have had difficulty in accessing documents mentioned as pre-registered. Please provide a correct link to the files.**

We apologize for the difficulties; the error was absolutely on our end. We had two OSF pages for the same project: one had our preregistration and the other had the rest of the files. The correct link to the pre-registration is: <https://osf.io/7ahks>, and has been updated throughout the manuscript. We have accessed this link on several separate computers, including in Incognito Mode, so we believe it should robustly work. We have now moved all datafiles to the correct OSF page (the OSF page associated with the preregistration above) and have updated the link throughout the manuscript: <https://osf.io/9pgkd/>.

**2) Please make sure to address the technical and statistical issues raised by both Reviewers such as multiplicity corrections and statistical trend analyses. In addition, please (a) state if data sets were tested for normality and which (parametric, nonparametric) statistical inference was used for respective sets, (b) which statistic (and directional or not) was used for each p-value, and (c) add variability measures where appropriate.**

We have addressed all statistical issues below.

**Reviewer #1**

**Reviewer #1: The authors have revised the manuscript to address the reviewer’s concerns, making the paper even stronger and clearer. The data set is large, but the authors do a good job of aligning the analysis of the data with their goals and distilling the main points of the findings. I appreciate the thoroughness of their response to the previous reviews. I have only a few very minor suggestions for the authors to take into consideration as they move forward with editing, to make the paper as clear as possible.**

We thank the reviewer for their kind words, and appreciate the remaining suggestions, which we have responded to below.

**Pg. 5-6: “because it elicited ratings only for developmental categories (e.g., “adolescents (ages 12-18)”),” This sentence is not complete.**

The sentence now reads “This study provides an exciting and promising window into understanding how gender stereotypes differ across the lifespan; however, because it elicited ratings only for developmental categories (e.g., “adolescents (ages 12-18)”), it does not allow us to draw conclusions about developmental trajectories.”

**Pg. 6: “in order for our general theories of gender stereotyping (e.g., Social Role Theory (e.g., Eagly & Wood, 2012; 2016) and the Stereotype Content Model; Fiske et al., 2002; Fiske et al., 1999) to be useful, they must fit the data not only for adults but also for children.” I’m not sure the authors want to imply these theories are not useful, as they were designed for stereotypes of adults and have created meaningful bodies of research. Certainly, it's possible they may not apply well to stereotypes of children (or it could be that the social roles of children do impact their gender stereotypes, as would be suggested by social role theory, which doesn't mean the stereotypes have to mirror adult stereotypes). So this sentence could be reworded to suggest these theories may need to be adapted or new theories created to address gender stereotypes across development, without throwing out these theories entirely. Otherwise, this seems like a strong point – to say these theories would not be useful if gender stereotypes differ across ages – that would need more elaboration and discussion that this one sentence.**

Thank you for pointing out this unfortunate wording. We do not wish to imply that the SCM isn’t useful. We now say: “First, in order for our general theories of gender stereotyping (e.g., Social Role Theory (e.g., Eagly & Wood, 2012; 2016) and the Stereotype Content Model; Fiske et al., 2002; Fiske et al., 1999) to be useful for understanding and predicting children’s learning about gender stereotypes, they must fit the data not only for adults but also for children”

**Pg. 12: The list of traits that are not prescriptive for gender includes three items that reference "kids" – gets pushed around by other kids, pulls other kids’ hair, thinks it’s funny when other kids are crying. It is unclear whether these items were used verbatim for all groups (even adolescents and adults) or whether the item changed to reflect the age group that was being rated (e.g., “gets pushed around by other adults”).**

Thank you for pointing out this ambiguity. These items were used verbatim.

**Given the multiple tests of comparison, it would be useful to include a statement at the beginning of the results that indicates the criterion for labeling something as a significant main effect or interaction (was it p < .05?). This may be stated in the preregistration, but the links provided for the preregistration took me only to the data and I did not see a preregistration document there (although perhaps I did not know where to look - but I suggest checking the link to make sure it leads to the preregistration documents). I know it is the effect size that qualifies a characteristics as a stereotype or not, but it is discussed how many traits showed significant effects, so knowing this cutoff is still relevant.**

We apologize again for the error in the preregistration link. Our alpha was .05, and all tests were two-tailed. Our effect-size cutoffs were also pre-registered.

**Pg. 17: The authors reference “In our previous work, we demonstrated that..” but do not give a citation (likely for blinding for review). I would suggest now adding in a citation here so that readers know what work is being referenced.**

Thank you. We have now added our citation.

**Table 6: Perhaps in the “direction switches” category it could be indicated which gender was higher at the youngest ages and then which was high later (the “switch”), instead of indicating “N/A.”**

When we attempted to implement this suggestion, we found that it looked quite clunky (e.g., “higher for girls at ages 1 and 4, and higher for boys for age 7+”). In addition, the analyses required to determine the precise nature of the “switch” are different from the analyses used in the rest of the table (the former involves pairwise comparisons within age, while the latter involves regression models over the entire dataset); we were concerned that reporting particular age information in this table might confuse readers.

However, we agree with the reviewer that “N/A” is not the ideal notation for this table. We now say “direction switches” in this column. Readers who are interested in understanding the precise nature of the switch can access our data and supplemental materials.

**Table 8 has blanks when the stereotype was not relevant to that age group, but Table 9 uses dashes (and one blank). Do these dashes also represent ages where the stereotype was not relevant? I would suggest using blanks in both tables as that seems the most intuitive (and I believe APA style suggests dashes when the data are not collected/irrelevant, which is not the case here).**

We have now made our formatting consistent, by changing all dashes to blanks.

**Reviewer #2: I was a reviewer on the prior submission. I really like the aim of this project – to descriptively catalogue gender stereotypes at a range of ages. The current manuscript is stronger and has overall addressed my comments well. I have a few comments that I hope will help to strengthen the paper further.**

Thank you! We really appreciate the time you’ve put into improving the manuscript.

**In response to my original comment about the pre-registration (#17), I appreciate the clarification, but I still can’t find the files. When I go to the OSF page, at first the only options available to me are “Files” “Wiki” and “Analytics”. Then when clicking “Analytics”, “Registrations” appears. But then when I click on “Registrations”, I get a message “there was an error loading this list”. So, I still have not been able to review the registrations.**

We are really sorry -- we (accidentally!) had two OSF pages for the same project with very similar titles; one had the data and the other had the preregistration. The correct preregistration link is now throughout the article, and we have moved all datasets etc… from the other OSF page to the correct one. As noted above, the correct link to the pre-registration is: <https://osf.io/7ahks> and the correct link the OSF page containing that pre-registration is: <https://osf.io/9pgkd/>

**I really appreciate the authors moving away from phrases like “lifespan” and being clearer in identifying the ages examined in this work. However, the phrase “across the developmental timespan” (e.g., as used in the abstract and the introduction) still implies that the developmental timespan encompasses only childhood / up to middle adulthood, when in fact development continues throughout the lifespan. Perhaps a phrase like “early lifespan” or “early developmental timespan” or “from childhood through early adulthood” would be more accurate?**

Thank you. We have changed it to “early developmental timespan” in most cases, and to the “developmental timepsan studied” when we are referring to the ages tested in our study.

**In the method: “For example, participants in one condition saw: “Today you will be**

**answering questions about how [common or typical/desirable] you think particular traits are among 1-year-old boys”.”Should either “common or typical” or “desirable” be listed, as it’s an example, or did participants within this condition actually rate on both commonness and desirability (which is what this wording could suggest)?**

It now says “...questions about how [“common or typical”][“desirable”] you think particular traits are among…”

**I really like the visualization for the interactions. But, only a linear term of age is used, which could potentially omit some characteristics that would have quadratic/cubic effects for one gender but not another. If there are nonlinear patterns of interaction e.g., if girls tend to increase and then decrease on a trait, whereas for boys it remains flat, that won’t necessarily appear in the interactions as specified if the overall (linear) change for girls is steady. It looks from the visualizations that for many of those characteristics that showed a difference in the linear slope (i.e., a significant interaction), there was likely some difference in quadratic/cubic change, too – e.g., childhood gender differences, adolescent gender differences – boost, and fluctuating gender differences. These traits just happen to have been identified by the linear interactions because there is also an overall difference in slope between genders, and not because the models run could identify these kinds of patterns. This suggests that there may be characteristics for which males and females differ in their quadratic/cubic patterns but not in their overall linear term. Could the authors re-rerun the regressions and see if including a quadratic or cubic term for age would suggest additional characteristics to explore? This can be done by adding both age(centered)^2 as a linear term and as interacting with gender, and age(centered)^3 as a linear term and as interacting with gender. This could potentially identify additional characteristics that fit the current set of visualization patterns, and/or new ones.**

We agree that this is a fascinating possibility, and one that is ripe for examination in future research. However, comparing models in these suggested ways would add a substantial amount of complexity and information to the paper, which is already dense. Adding to the complexity is that these analyses would not only be exploratory, but also atheoretical: while we acknowledge that there are some visible non-linearities in our data, we know of no theories that predict a particular cubic or quadratic effect of age -- or, critically, an interaction of target gender with a cubic or quadratic age function. This makes it challenging to explore this question in a targeted way -- when conducting exploratory analyses that deviate from the plan, it is best practice to do so when theoretically motivated.

More generally, our ability to make fine-grained determinations about the shape of developmental change hinges on the density of our age sampling; while our sampling of different ages across childhood allows us to make general statements about the overall trajectory, without fine-grained sampling of ages (i.e., at least of every year), any descriptions of the shape of developmental change will be impacted substantially by our sampling frequency (for an excellent description and demonstration of this in the domain of motor development, see Adolph et al., 2008).

Our goal is to provide some preliminary observations about trends in these data, and then make the dataset available for other researchers to examine in numerous different ways. As such, we have added the sentence to the General Discussion on pp. 31-32. :

“Further, a visual inspection of our data suggests the possibility that some of the developmental changes in gender stereotyping may be non-linear. The present study was not designed to differentiate possible developmental trajectories and did not sample ages densely enough to effectively do so (e.g., step-functions vs. logarithmic vs. quadratic vs. linear; see Adolph, 2008 for review). Thus, we encourage researchers to explore our dataset, and to conduct further research specifically aimed at detecting differences in the shape of change of gender stereotyping across the lifespan. In addition, we describe some of the qualitative patterns that emerged in our dataset below”

**Regarding: “Critically, these findings are unlikely to be due to biases in the characteristics tested in our study: if they were, we would not expect the symmetrical rate of descriptive stereotypes.” (p. 28). I don’t quite follow this, can the authors provide a little more explanation of the logic here?**

Upon revisiting that paragraph, we also are unsure what we intended with this sentence -- we have removed it from the manuscript.
